# Search for cardiac calcium cycling gene mutations in familial ventricular arrhythmias resembling catecholaminergic polymorphic ventricular tachycardia

**DOI:** 10.1186/1471-2350-10-12

**Published:** 2009-02-12

**Authors:** Annukka Marjamaa, Päivi Laitinen-Forsblom, Annukka M Lahtinen, Matti Viitasalo, Lauri Toivonen, Kimmo Kontula, Heikki Swan

**Affiliations:** 1 Research Program in Molecular Medicine, University of Helsinki, Helsinki, Finland; 2 Department of Medicine, University of Helsinki, Helsinki, Finland; 3 Department of Cardiology, University of Helsinki, Helsinki, Finland

## Abstract

**Background:**

Catecholaminergic polymorphic ventricular tachycardia (CPVT) is a severe inherited cardiac disorder caused by mutations predominantly in the ryanodine receptor (*RyR2*) gene. We sought to identify mutations in genes affecting cardiac calcium cycling in patients with CPVT and in less typical familial exercise-related ventricular arrhythmias.

**Methods and Results:**

We recruited 33 consecutive patients with frequent ventricular premature complexes (VPCs) without structural heart disease and often history of syncope or sudden death in family. Sixteen of the patients featured a phenotype typical of CPVT. In 17 patients, VPCs emerged also at rest. Exercise stress test and echocardiography were performed to each patient and 232 family members. Familial background was evident in 42% of cases (n = 14). We sequenced all the coding exons of the *RyR2*, *FKBP1B*, *ATP2A2 *and *SLC8A1 *genes from the index patients. Single channel recordings of a mutant RyR2 were performed in planar lipid bilayers. Two novel *RyR2 *missense mutations (R1051P and S616L) and two *RyR2 *exon 3 deletions were identified, explaining 25% of the CPVT phenotypes. A rare variant (N3308S) with open probabilities similar to the wild type channels *in vitro*, was evident in a patient with resting VPCs. No disease-causing variants were detectable in the *FKBP1B*, *ATP2A2 *or *SLC8A1 *genes.

**Conclusion:**

We report two novel CPVT-causing *RyR2 *mutations and a novel *RyR2 *variant of uncertain clinical significance in a patient with abundant resting VPCs. Our data also strengthen the previous assumption that exon 3 deletions of *RyR2 *should screened for in CPVT and related phenotypes.

## Background

Catecholaminergic polymorphic ventricular tachycardia (CPVT) is a severe, autosomal dominantly inherited disorder characterized by stress-induced polymorphic ventricular tachycardias in a structurally normal heart [1-3]. Thus far, the ryanodine receptor type 2 (*RyR2*) and calsequestrin (*CASQ2*) genes have been identified to underlie the disease. Yet, many patients with clinical features comparable with CPVT harbor no apparent mutations in these genes, thus reflecting the existence of yet unidentified non-coding mutations or genetic heterogeneity of this disorder.

Mutations or variants in calstabin (*FKBP1B*), sarcoplasmic reticulum Ca^2+^-ATPase (*ATP2A2*) or sodium-calcium exchanger (*SLC8A1*) genes have until now not been reported to be associated with increased risk of human ventricular tachyarrhythmias. Although still a matter of controversy [4], a number of delicate *in vitro *studies suggest that depletion of calstabin, which normally stabilizes the RyR2 channel complex, is related to CPVT pathogenesis [3,5,6]. SERCA2a (Ca^2+^-ATPase), an ion pump located on the sarcoplasmic reticulum (SR) in a cardiac myocyte, plays a pivotal role in transporting intracellular Ca^2+ ^ions back to the SR in the beginning of diastole. Thus far, SERCA2a encoded by the *ATP2A2 *gene has been the target of extensive *in vitro *and *in vivo *experiments in heart failure only [7-10], whereas a mutated cardiac sodium-calcium exchanger 1 (NCX1), the major Ca^2+ ^ion remover in cardiac myocyte, has been shown to cause cardiac fibrillation in embryonic zebrafish hearts [11,12].

According to several investigators, circulating catecholamines cause the abnormal activation of RyR2 channels and thus an arrhythmogenic leak of Ca^2+ ^ions from the sarcoplasmic reticulum [13]. However, it has been hypothesized that *RyR2 *mutations could lead to channel dysfunction also in the absence of sympathetic stimuli [14]. In this study, we aimed to broaden the knowledge of disease-causing *RyR2 *mutations in resting and exercise-induced VPCs and to elucidate the involvement of not only *RyR2*, but also three other candidate genes in the pathology of ventricular tachyarrhythmias. We hypothesized that FKBP12.6, SERCA2a and NCX1, as integral proteins in cardiac Ca^2+ ^signaling, might play a role in the pathogenesis of human ventricular arrhythmias and which defects might manifest with clinical features resembling CPVT.

## Methods

### Patients and controls

We recruited 33 consecutive symptomatic patients referred to Helsinki University Hospital for genetic studies of cardiac ion channel disorders because of frequent ventricular premature complexes (VPCs) occurring during exercise and consecutive VPCs, history of syncope and/or sudden juvenile death in family. None had apparent structural heart disease or prolonged QT interval. A total of 16 patient featured arrhythmias typical of CPVT in the exercise stress test. The remaining 17 subjects had frequent VPCs and often salvos of VPCs also at rest, thus differing from CPVT phenotype. A health enquiry, a 12-lead resting ECG, an exercise stress test, a cardiac ultrasonography and 24-hour ambulatory ECG recording were registered from each index patient. If over ten VPCs were evident in the exercise stress test of patient's first degree relatives, the clinical examinations including 12-lead resting ECG, exercise stress test and cardiac ultrasonography were extended to available second-degree relatives. A total of 232 relatives were clinically evaluated in the course of the study. History regarding the age of onset, episodes of syncopal spells and occurrence of sudden juvenile (≤ 40 years) death in the patient's first and second degree relatives were collected. The control DNA samples of 300 apparently healthy blood donors were provided by the Finnish Red Cross Blood Service. An informed consent was obtained from each patient and the Ethics Committee of the department approved the study.

### DNA analyses

We sequenced all the 105 coding exons and exon-intron boundaries of *RyR2 *(NM_001035) gene using ABI 3730 Automatic DNA sequencer (Applied Biosystems, Foster City, MA, USA) provided by the National Public Health Institute of Finland. Reference sequences were obtained from the National Centre for Biotechnology Information (NCBI) database. The primer sequences, designed mainly by N. Tiso, and reaction conditions for PCR amplification are available by contacting the corresponding author. The previously reported 1.1 kb *RyR2 *exon 3 deletion was investigated using multiplex ligation-dependent probe amplification (MLPA) analysis of *RyR2 *exon 3 (Salsa MLPA Kit P168, MRC Holland, Amsterdam, the Netherlands) and with PCR using primers CACAGAACAGGACCAAGTTAGAGG (forward) in intron 2 and CATTACCTTCCTGACACACTTCAT (reverse) in intron 3 of the *RyR2 *gene [15]. When present, the architecture of the deletion was investigated in more detail by DNA sequencing. A functional candidate gene approach was applied to probands that failed to present *RyR2 *mutations. These indexes were additionally screened for variations in the exonic regions of *FKBP1B *(NM_004116), *ATP2A2 *(NM_170665) and *SLC8A1 *(NM_021097) genes coding for the integral proteins in cardiac Ca^2+ ^signaling. CPVT caused by mutations in the *CASQ2 *gene is proposed to follow the autosomal recessive inheritance, while the disease phenotype in our patients with a familial disorder, followed autosomal dominant pattern. In addition, sequencing of the *CASQ2 *gene was excluded from the study protocol because our previous study on *CASQ2 *failed to detect any rare variants in Finnish CPVT patients [16]. Primer sequences and the conditions for sequencing the three genes are obtainable by contacting the authors. Detection of observed allelic *RyR2 *variants in family members and 300 healthy controls was achieved by denaturing High Performance Liquid Chromatography (dHPLC, WAVE Nucleic Acid Fragment Analysis System HSM 3500A). Direct sequencing confirmed the genomic variations leading to divergent chromatogram profiles. The detection of *ATP2A2 *T982M in the relatives and controls was achieved by restriction analysis utilizing the Lwel enzyme (Fermentas International Inc, Burlington, Ontario, Canada).

### *In vitro *electrophysiological studies

RyR2 N3308S mutagenesis was performed with QuikChange II XL mutagenesis kit (Stratagene, La Jolla, CA, USA) using cassettes of hRyR2 subcloned into pBlueScript vector. Mutated fragments of hRyR2 were cloned back into full-length pCMV5/hRyR2 with FseI and NheI. HEK293 cells were co-transfected with 10 μg of pCMV5/hRyR2 and an equal molar amount of pCMV5/FKBP12.6 per T175 flask using calcium phosphate precipitation technique.

Single channel recordings were conducted in planar lipid bilayers under voltage clamp conditions. Microsomes containing the recombinant RyR2 channels were added to the *cis*-chamber and fused to the lipid bilayer consisting of 3:1 phosphatidyl ethanolamine/phosphatidyl serine (Avanti Polar Lipids, Alabaster, AL, USA). Fusion was induced by adding KCl to the *cis*-chamber, and the created ion gradient was eliminated after fusion by perfusing the chamber with *cis*-solution. The bathing solutions were as follows: *cis *solution, 50 mM KCl, 250 mM HEPES, 125 mM TRIS, 1 mM EGTA, 0.5 mM CaCl_2_; *trans*-solution 50 mM KCl, 53 mM Ba(OH)_2 _and 250 mM HEPES. All experiments were carried out in pH 7.35, and free Ca^2+ ^concentration was determined using the program CHELATOR. 20 mM CaCl_2 _was added sequentially to the *cis*-side, and the channel activity was recorded for at least 3 min in each concentrations. At the end of each experiment, ryanodine was applied to confirm the class of the channel. Data were collected using AxoScope1 (Axon Instruments, Foster City, CA, USA) and analyzed by pClamp 6 program (Axon Instruments). The open probability (P_0_) was identified by 50% threshold analysis.

### Statistical analyses

Statistical analysis was performed with student's t-test for continuous variables and with *X*^2 ^and Fischer's exact test for dichotomous variables using the SPSS 13.0 software. P-value of < 0.05 was considered statistically significant.

## Results

### Clinical studies

**Figure 1:**
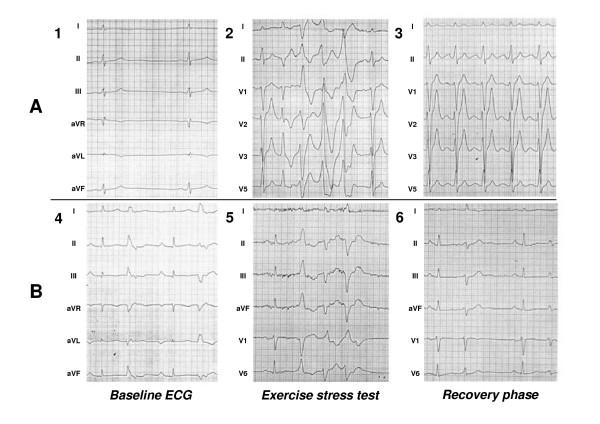
**Sample ECG recordings from the exercise stress tests in the Group A and B**. A 33-year-old male patient from Group A reported exercise-induced syncope since the age of 14. Resting ECG was normal except for a relative bradycardia of 45 bpm (1). After the threshold heart rate of 128 bpm, the exercise stress test revealed polymorphic VPCs typical of CPVT (2) that disappeared in the recovery phase (3). Three of the 16 examined individuals in the family had a similar phenotype and carried the *RyR2 *exon 3 deletion. The 57-year-old female patient in Group B showed frequent VPCs in baseline ECG (4), in the exercise stress test (5) and in the recovery phase (6). A total of 13 patients of the 77 clinically evaluated subjects in the family featured a similar atypical phenotype. No disease-causing variants were detectable in the screened genes.

**Figure 2:**
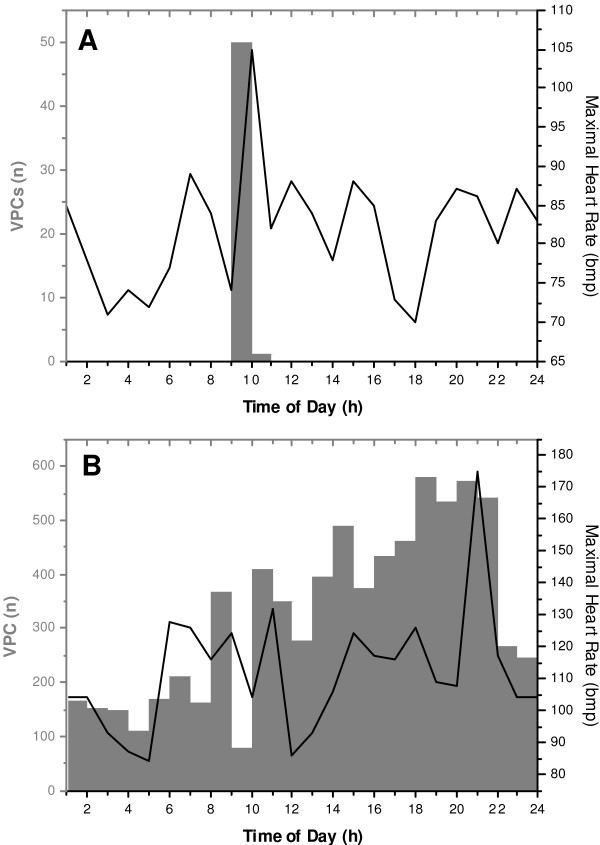
**Sample 24-hour ambulatory ECG recordings in the Group A and B**. The 24-hour ECG recording revealed a number of exercise-provoked VPCs (n = 51) occurring solely above the heart rate of 90 bpm in a typical CPVT patient from group A. In a patient from group B, VPCs (n = 8 042) occurred also in resting conditions. The number of VPCs per hour is indicated by gray bars. The maximal heart rate per hour is shown by the curves. ECG = electrocardiogram, VPC = ventricular premature complex, bpm = beats per minute.

VPCs appeared in 16 cases solely upon physical exercise but not in resting conditions thus resembling the arrhythmias of classical catecholaminergic polymorphic ventricular tachycardia (Group A) [1,17,18]. In seventeen of the 33 patients, frequent VPCs were also observed at rest and at the recovery phase of the exercise stress test (Group B) as illustrated by an exemplary ECG recording (Figure 1) and by bar graphs summarizing the number of VPCs per hour in the 24 h Holter recordings (Figure 2). The clinical data from the subjects are summarized in Table 1. In the two groups, the mean age of onset was 26 ± 17 years. In the exercise stress tests, the number of successive VPCs was greater in the CPVT phenotype, but the difference was not statistically significant between the two groups (p = 0.08). The probands in the group B had statistically significantly more VPCs (p < 0.001) and more consecutive ventricular complexes (p < 0.01) than the CPVT patients in the 24 h ECG recordings. A total of six probands (38%) in the group A had relatives featuring similar arrhythmias in the clinical evaluation, whereas in the group B, seven probands (41%) had affected relatives. In group A, there were more syncopal spells and sudden juvenile deaths in the first and second-degree relatives, but the difference to group B was not statistically significant. In both phenotypes, one index patient had suffered from juvenile (≤ 40 years) sudden cardiac death.

**Table 1:** Clinical Characteristics of the Affected Individuals

		CPVT (Group A)	Resting VPCs (Group B)	*P*
n		16	17	NS
Females, n (%)		6 (38)	13 (77)	<0.05
Age at onset, y		25 ± 16 (10–64)	27 ± 18 (5–51)	NS
Syncope, n (%)		14 (88)	9 (53)	NS
Heart rate, bpm		60 ± 15 (38–98)	73 ± 20 (42–99)	NS
QT, ms		430 ± 55 (350–550)	405 ± 55 (320–490)	NS
QTc, ms		420 ± 25 (380–440)	430 ± 30 (380–450)	NS
Exercise stress test	Threshold heart rate, bmp	127 ± 30 (87–184)	-	-
	Consecutive VPCs, n	4 ± 6 (0–15)	1 ± 1 (0–5)	NS
24 hour ECG recording	VPCs, n	560 ± 800 (0–2670)	13800 ± 11100 (224 – 33200)	<0.001
	Consecutive VPCs, n	1 ± 1 (0–3)	5 ± 6 (0–24)	<0.01
Familial occurrence, n (%)		6 (38)	7 (41)	NS
Affected relatives, n (%)		12 (16)	26 (17)	NS

SD in family, n (%)		5 (31)	3 (18)	NS

Values are mean ± SD (range). VPCs, ventricular premature complexes; ECG, electrocardiogram; SD, history of sudden death at age ≤ 40 in the first or second degree relatives; NS, Statistically non-significant.

### Molecular genetic studies

#### Identification of *RyR2 *missense mutations

**Figure 3:**
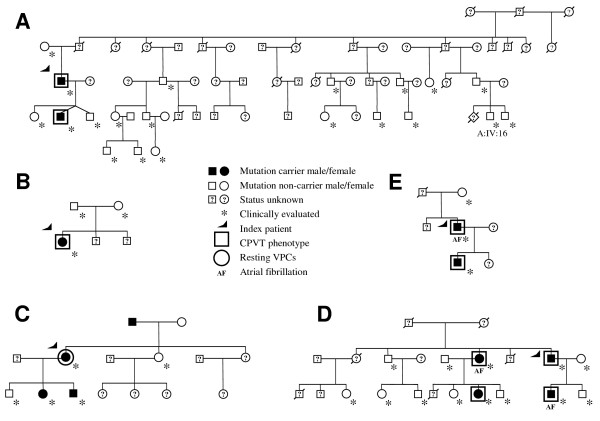
**The pedigrees of the families carrying *RyR2 *R1051P (A), *RyR2 *S616L (B), *RyR2 *N3308S (C) and the *RyR2 *exon 3 deletions (D,E)**. The carrier status of the mutation is illustrated by black circles and squares. Clinically evaluated family members are shown with an asterisk. Open large squares present clinically affected individuals with CPVT phenotype, while open large circle symbolizes the disease phenotype of frequent VPCs also in resting conditions. Deceased individuals are indicated with a slash.

Direct DNA sequencing of the 105 coding exons of *RyR2 *gene revealed two novel missense mutations (R1051P and S616L) in the group A [see Additional file 1]. The mutations were located in evolutionarily conserved amino acids [see Additional file 1] and could not be detected in any of the 300 blood donors. The index patient carrying *RyR2 *R1051P mutation (Figure 3A) was 35 years old and had experienced several syncopal spells since the age of 30. Lateral ST-alterations typical of left ventricular hypertrophy emerged in resting ECG, but the echocardiography revealed only a minor septal thickening up to 13 mm. The son of the patient carrying the *RyR2 *R1051P mutation had a similar phenotype, but the onset was at the early adolescence and the exercise stress test performed thereafter showed exercise-provoked VPCs characteristic for CPVT. Furthermore, no morphological alterations could be observed in the resting ECG or in the echocardiography. A son of the index's cousin (A:IV:16 in Figure 3A), aged 19 years, received a diagnosis of hypertrophic cardiomyopathy with septal thickening up to 20 mm and a 20 mmHg – gradient in the outflow tract. No cardiac arrhythmias were evident, and the patient lacked the R1051P mutation. The rest of the family (n = 17) featured no abnormalities in the clinical evaluation and did not carry the familial *RyR2 *defect.

The S616L mutation (Figure 3B) occurred in a female adolescent with exercise-induced syncopal spells since the age of twelve. Resting ECG showed a partly ectopic rhythm, and exercise stress test revealed polymorphic VPCs in a series of several beats. The mutation appeared to arise *de novo *as the parents of the index featured no pathological alterations in their exercise stress tests nor carried the *RyR2 *S616L.

In the group B, a novel *RyR2 *variation N3308S was discovered, but it was also detectable in one of the 600 control alleles (Figure 3C). The proband was a 34-year-old woman first presenting with palpitations and presyncopal episodes due to right ventricular outflow tract arrhythmias. Holter recordings uncovered over 20,000 predominantly single VPCs, but also bigeminial cycles and salvos up to four successive beats. The coronary angiography showed normal coronary arteries. The father of the index patient carried the variant but was not available for clinical evaluation. Two of the three descendants reported episodes of syncope and carried the same variant, yet their exercise stress tests with less than ten single monomorphic VPCs were judged to be within normal range.

#### Identification of *RyR2 *exon 3 deletions

An exon 3 deletion, approximately 1.1 kb in size, was identified in two (13%) separate CPVT families. In Family D (Figure 3), the 33-year old index patient featured syncopal spells and frequent bidirectional VPCs during exercise at the time of diagnosis. His son, sister and niece featured a similar symptomatic CPVT phenotype. During follow-up of over 25 years, the index patient's son and sister showed paroxysmal atrial fibrillation. The sister also featured sinusbradycardia and atrioventricular conduction abnormalities, whereas only bradycardia was observed in her affected daughter. Apart from the ascending aorta dilatation (up to 57 mm) in the index patient's sister, none of the affected subjects showed major structural cardiovascular abnormalities. The analyses of the available DNAs revealed a 1.1 kb deletion of the *RyR2 *gene (c.168-301_c.273+722del1128) eliminating the highly conserved exon 3.

In the Family E (Figure 3), the index patient aged 39 years featured presyncopal spells, atrial fibrillation, bradycardia and multifocal VPCs up to runs of ventricular tachycardia upon exercise stress test. In the follow-up, he showed increased trabeculation of the left ventricle suggestive of non-compaction cardiomyopathy. His son featured polymorphic VPCs upon exercise in the absence of atrial fibrillation. A molecular analysis of the *RyR2 *exon 3 deletion revealed the previously reported [15] 1.1 kb base deletion (c.168-228_c.273+793del1126) in these two patients of Family E. Both types (Family D, Family E) of genomic *RyR2 *deletions, although slightly different in their nature, are predicted to result in an identical in-frame deletion of 35 amino acids (p.Asn57-Gly91) from the *RyR2 *protein.

#### Other genes affecting cardiac calcium signaling

Screening for mutations in *FKBP1B *and *SLC8A1 *genes did not reveal any amino-acid alterations. A novel, conserved polymorphism T982M in the *ATP2A2 *gene was detectable in four (12%) individuals, two in both subgroups, and it was also identifiable in 10 of the 291 (3%) control individuals (p = 0.07).

### *In vitro *electrophysiological studies

**Figure 4:**
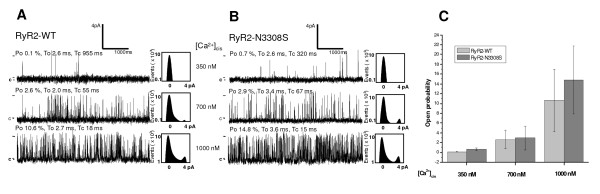
**The representative traces of single channel recordings of the RyR2 WT (A) and the RyR2 N3308S (B) at cytosolic Ca^2+ ^concentrations of 350 nM, 700 nM and 1 μM**. The channel openings are upward deflections. The open probabilities (Po) of the recorded channels are shown in the bar graph (C). No statistically significant differences were observable in the sensitivity of the channels to the cytosolic Ca^2+ ^concentrations. Po = open probability, To = mean open time, Tc = mean closing time, c = closed state.

Since the *RyR2 *N3308S mutation was associated with an atypical clinical phenotype, it was considered pertinent to study it in more detail *in vitro*. The representative data from the single channel recordings of the wild type RyR2 and N3308S are shown in Figure 4. The wild type RyR2 (n = 8) featured a mean open probability (Po) of 0.1 ± 0.1% at 350 nM cytosolic Ca^2+ ^concentration, 2.6 ± 1.9% at 700 nM and 10.6 ± 6.4% at upper systolic 1 μM cytosolic Ca^2+ ^concentration (A). The measurements of RyR2 N3308S (n = 8) showed a mean Po of 0.7 ± 0.3% at 350 nM, 2.9 ± 2.4% at 700 nM and 14.8 ± 6.9% at 1 μM cytosolic Ca^2+ ^concentration (B). The open probabilities of the mutant channel did not differ statistically significantly from the wild type over the range of 90 nM free-[Ca^2+^]_cis _to maximal activity. In addition, the kinetics, mean open time (T_o_) and mean closed time (T_c_) of the channels were analogous.

## Discussion and conclusion

Despite marked progress in understanding the pathogenesis of inherited ventricular arrhythmias, the molecular genetics of CPVT and frequent ventricular premature complexes without structural heart disease is incompletely known. In the present study, two CPVT families carried a corresponding in-frame *RyR2 *exon 3 deletion that has recently been characterized by Bhuiyan et al in two kindred of exercise-related ventricular arrhytmias, atrial arrhythmias, conduction defects and left ventricular dysfunction [15]. Previously, it was shown that the genomic deletion of the *RyR2 *exon 3 occurred as a result of a *Alu*-repeat mediated polymerase slippage during chromosomal replication [15]. Neither of the families in the present study featured consistent structural abnormalities suggesting that the deleted *RyR2 *exon 3 may underlie a spectrum of a purely electric CPVT disorder to a more complex disorders as shown previously [15]. Characterization of the large *RyR2 *exon 3 deletion independently in altogether four unrelated families with CPVT-related disorders thus far suggests that this region may be of notable importance in the pathogenesis of inherited arrhythmia disorders.

In the present study, four (25%) out of the 16 probands featuring typical CPVT carried a disrupted *RyR2 *gene. In our previous studies, a *RyR2 *defect has been evident in an average of 40% of CPVT patients [18,16] similarly to other patient populations [13]. As we sequenced the 105 exons of the *RyR2 *gene and analyzed the DNAs for exon 3 deletions, our study suggests existence of non-coding intron mutations, other large yet unidentified genomic deletions, alterations in the regulatory units or genetic heterogeneity of the disorder. Combining present data to our previous studies, *RyR2 *alterations explain 35% of the CPVT phenotype in the Finnish probands (n = 26) of which 2 out of 9 (22%) appeared to be *de novo *mutations. The low frequency of *de novo *mutations calls for systematic cardiovascular examinations for all first degree relatives. In addition, the identified *RyR2 *S616L and R1051P missense mutations are expected to extend the N-terminal hot-spot region of the *RyR2 *gene which further challenges the method of targeted mutational analysis in which only previously mutation positive exons are sequenced.

Molecular genetic approaches should be applied in gaining further understanding in familial VPCs that occur also in resting conditions. Recently, several *in vitro *studies have questioned the occurrence of arrhythmias in CPVT merely in the presence of adrenergic stimulus [14,19]. In our study, only one index showing resting VPCs carried a variant in the *RyR2 *gene. According to the proposed criteria for high risk right ventricular outflow tract – arrhythmias [20], this phenotype with extremely frequent VPCs and a history of syncope was of malignant nature. However, the asparagine substitution to serine at position 3308 maintains the polarity of the amino acid and results in a neutral replacement. In addition, the N3308S was detected in one of the 300 apparently healthy controls. The single channel experiments carried out in planar lipid bilayers did not show enhanced sensitivity of the mutant channel to the cytosolic Ca^2+ ^under basal conditions. Collectively, the evidence of the causal role of this variant is unconvincing and suggests that the *RyR2 *N3308S is an innocent variant despite its rare occurrence in the Finnish population. However, a search for the variant in patients with a similar phenotype may be justifiable in other populations.

Despite the lack of convincing disease-causing *RyR2 *mutations in our patients with VPCs at rest, the occurrence of syncopal spells and the proportion of familial occurrence (38% vs. 41%) did not differ between the CPVT and resting VPC phenotype. Furthermore, the similar incidence of sudden juvenile death among the first and second degree relatives of the two groups suggests that even this phenotype with resting VPCs may be malignant. Elucidating the genetic background in this heterogeneous group is fundamental so that the malignant forms of the disorder could be identified. In the present study, the limited sample size precludes any definitive statements of the occurrence of rare mutations in these candidate genes in other study populations. In addition, the lack of information concerning the *in vitro *functional properties of the mutant *RyR2 *S616L and R1051P channels remains as a limitation of the present study. We conclude that mutations in *FKBP1B*, *ATP2A2 *and *SLC8A1 *appear subsidiary in the pathogenesis of frequent VPCs of unknown etiology in Finnish patients. *RyR2 *involvement in potentially malignant frequent resting VPCs requires further clarification. Large genomic *RyR2 *deletions involving exon 3 provide a target for future genetic studies in disorders resembling CPVT.

## Competing interests

The authors declare that they have no competing interests.

## Authors' contributions

AM has participated in the designing of the present survey, performed genotyping and *in vitro *experiments, conduced the data analysis and drafted the manuscript. PL-F has participated in the designing of the present survey, participated in the genotyping process and in writing of the manuscript. AML has participated in the data production. MV has participated in the data collection, data analysis and in writing of the manuscript. LT has participated in the designing of the present survey, data analysis and in writing of the manuscript. KK has participated in the designing of the present survey, data analysis and in writing of the manuscript. HS has participated in the designing of the present survey, data collection, data analysis and in writing of the manuscript. All authors have read and approved the final manuscript.

## Pre-publication history

The pre-publication history for this paper can be accessed here:



## Supplementary Material

Additional file 1 **The nucleotide sequences traces and the conservation of the three *RyR2 *mutations.** The data provided represent the nucleotide sequences traces and the conservation profiles of the three identified *RyR2 *mutations. Click here for file
