# Supplementary material for: Search for cardiac calcium cycling gene mutations in familial ventricular arrhythmias resembling catecholaminergic polymorphic ventricular tachycardia
Source: BMC Med Genet. 2009 Feb 12;10:12. doi: 10.1186/1471-2350-10-12 (PMC2667497; doi:10.1186/1471-2350-10-12)
Supplement: Additional file 1 — The nucleotide sequences traces and the conservation of the three RyR2 mutations. The data provided represent the nucleotide sequences traces and the conservation profiles of the three identified RyR2 mutations. [file 1471-2350-10-12-S1.doc]

Additional files

Additional file 1

The nucleotide sequences traces and the conservation of the three *RyR2* mutations
